# The Muscle Ankyrin Repeat Proteins CARP, Ankrd2, and DARP Are Not Essential for Normal Cardiac Development and Function at Basal Conditions and in Response to Pressure Overload

**DOI:** 10.1371/journal.pone.0093638

**Published:** 2014-04-15

**Authors:** Marie-Louise Bang, Yusu Gu, Nancy D. Dalton, Kirk L. Peterson, Kenneth R. Chien, Ju Chen

**Affiliations:** 1 Institute of Genetic and Biomedical Research, UOS Milan, National Research Council and Humanitas Clinical and Research Center, Rozzano (Milan), Italy; 2 Department of Medicine, University of California San Diego, La Jolla, California, United States of America; 3 Department of Cell and Molecular Biology and Medicine, Karolinska Insititutet, Stockholm, Sweden; 4 Harvard University, Department of Stem Cell and Regenerative Biology, Cambridge, Massachusetts, United States of America; Mayo Clinic, United States of America

## Abstract

Ankrd1/CARP, Ankrd2/Arpp, and Ankrd23/DARP belong to a family of stress inducible ankyrin repeat proteins expressed in striated muscle (MARPs). The MARPs are homologous in structure and localized in the nucleus where they negatively regulate gene expression as well as in the sarcomeric I-band, where they are thought to be involved in mechanosensing. Together with their strong induction during cardiac disease and the identification of causative *Ankrd1* gene mutations in cardiomyopathy patients, this suggests their important roles in cardiac development, function, and disease. To determine the functional role of MARPs *in vivo*, we studied knockout (KO) mice of each of the three family members. Single KO mice were viable and had no apparent cardiac phenotype. We therefore hypothesized that the three highly homologous MARP proteins may have redundant functions in the heart and studied double and triple MARP KO mice. Unexpectedly, MARP triple KO mice were viable and had normal cardiac function both at basal levels and in response to mechanical pressure overload induced by transverse aortic constriction as assessed by echocardiography and hemodynamic studies. Thus, CARP, Ankrd2, and DARP are not essential for normal cardiac development and function at basal conditions and in response to mechanical pressure overload.

## Introduction

In cardiac and skeletal muscle, a family of muscle specific ankyrin repeat proteins (MARPs) includes cardiac ankyrin repeat protein (CARP/Ankrd1) [1], [2], [3], [4], Ankyrin repeat domain protein 2 (Ankrd2/Arpp) [5], [6], and diabetes related ankyrin repeat protein (DARP/Ankrd23) [7]. The three proteins share ∼50% sequence identity and all contain a N-terminal nuclear localization signal and four ankyrin repeats [8]. CARP is mainly expressed in cardiac muscle, Ankrd2 in skeletal muscle, and DARP at similar amounts in both cardiac and skeletal muscle; however all three proteins can be induced both in heart and skeletal muscle in response to various forms of stress. All three MARPs are localized in the nucleus as well as the sarcomeric I-band where they bind to the titin N2A region between the two principal spring elements, the tandem immunoglobulin (Ig) and PEVK spring regions, suggesting their role in stretch/stress sensing [8].

Cardiac ankyrin repeat protein (CARP) was first identified as a cytokine-inducible gene in microvascular endothelial cells [2] and later recognized as a cardiac doxorubicin responsive nuclear transcription factor downstream of the Nkx2.5 pathway [3], [4]. CARP is highly expressed in the early embryonic heart and is downregulated to lower levels in adult heart. However, during postnatal hypertrophy induced by mechanical pressure overload or α1-and β-adrenergic agonist stimulation in animal models, CARP is markedly induced, suggesting that it is a member of the hypertrophic embryonic gene program [9], [10], [11], [12], [13], [14], [15]. Likewise, CARP is induced in patients with hypertrophic, dilated, ischemic, and arrhythmogenic right ventricular cardiomyopathy [16], [17], [18] and missense mutations in the *Ankrd1* gene were recently shown to be causative for human dilated and hypertrophic cardiomyopathy [19], [20], [21]. Further evidence supporting an important role of CARP in regulating the cardiac hypertrophic response comes from a recent study of cardiac-specific CARP-overexpressing transgenic mice, which were found to show an attenuated cardiac hypertrophic response following mechanical pressure overload or isoproterenol infusion [22]. In *in vitro* studies, CARP has been shown to bind to the ubiquitous Y-box transcription factor 1 (YB-1) and act as a nuclear transcriptional cofactor by negatively regulating the expression of cardiac genes, including the ventricular specific myosin light chain-2v, atrial natriuretic factor, and cardiac troponin C [3], [4], [12]. Furthermore, knockdown of CARP in rat cardiomyocytes resulted in sarcomere disarray and inhibition of myofilament gene transcription [23]. CARP can bind to both the titin N2A region and myopalladin in the I-band, suggesting its role in mechanosensing and regulation of gene expression in response to muscle stress [8], [24]. In addition, CARP has been shown to be able to dimerize and bind to desmin, the muscle specific RING finger proteins MuRF1 and MuRF2, cardiac calsequestrin 2 [25], [26], [27], and the tumor suppressor protein p53 [28], suggesting a versatile role of CARP in the heart.

Ankyrin repeat protein 2 (stretch responsive-muscle) (Ankrd2)/Ankyrin Repeat protein with PEST and Proline-rich region (Arpp) was first described by Kemp as a striated muscle specific gene induced by mechanical stretch and involved in mechanically-induced skeletal muscle hypertrophy [5]. Further studies revealed that Ankrd2 is selectively expressed in type I fibers of skeletal muscle muscle [29], [30] and present only at low levels in the left and right ventricles, the interventricular septum, and the apex of the heart [6]. During fetal development Ankrd2 is diffusely expressed in skeletal muscles and is barely detectable in the heart [29]. Like CARP, Ankrd2 is highly responsive to acute stress and has been found to be upregulated during myoblast differentiation [29] and in response to mechanical muscle stretch [5], [31], exercise [32], eccentric contractions [33], [34], and denervation [35]. Furthermore, Ankrd2 has been shown to translate to the nuclei in myofibers in the vicinity of injured fibers [36]. In human, Ankrd2 is upregulated in congenital myopathies [37] and downregulated in muscular dystrophy [30], [37] and show an altered expression pattern in spinal muscular atrophy and amylotrophic lateral sclerosis [30], [37], [38], likely due to changes in fiber type distribution [39]. Furthermore, Ankrd2 was found to be upregulated in human dilated cardiomyopathy [16], suggesting that Ankrd2 may also be involved in cardiac pathologies. In addition to the titin N2A domain, Ankrd2 has been shown to interact with T-cap/telethonin as well the transcription factors YB-1, promyelocytic leukemia protein (PML), and p53, suggesting that Ankrd2 may also be involved in muscle gene regulation [40]. Furthermore, our recent results have demonstrated that Akt2-phosphorylated Ankrd2 can bind directly to the NF-kB p50 subunit and negatively regulate inflammatory responses during muscle differentiation in response to oxidative stress [41].

Diabetes-related ankyrin repeat protein (DARP) is expressed in heart, skeletal muscle, and brown adipose tissue [7] and is the least studied of the three proteins. It was found to be upregulated in the hearts of type 2 diabetic and insulin resistant mice and to show altered expression after metabolic challenge, suggesting its potential role in energy metabolism [7].

Taken together, several lines of evidence suggest that the MARPs belong to a family of stress responsive proteins, which plays critical roles in the heart. However, although the MARPs have been extensively studied, their functional role in the heart *in vivo* remains unknown. To address this, we studied knockout (KO) mice for each of the three MARP family members. All three KO mouse models are viable and have no apparent cardiac phenotype, which we hypothesized might be due to redundant functions of the highly homologous MARP proteins. We therefore studied double (CARP/Ankrd2, CARP/DARP, and Ankrd2/DARP) and triple (CARP/Ankrd2/DARP) MARP KO mice, but to our surprise both double and triple KO mice were viable and extensive analyses up to 17 months of age did not reveal any abnormalities in cardiac development or function, either at baseline or in response to biomechanical stress induced by pressure overload hypertrophy. Thus, our results suggest that CARP, Ankrd2, and DARP are not required for normal cardiac development and function at basal conditions and in response to mechanical pressure overload *in vivo*.

## Materials and Methods

### Ethics Statement

All animal procedures were approved by the University of California San Diego Animal Care and Use Committee (Approval reference number: S01049) and performed in full compliance with the guidelines of the Guide for the Care and Use of Laboratory Animals published by the US National Institutes of Health (NIH Publication No. 85-23, revised 1996). Special attention was paid to animal welfare and to minimize the number of animals used and their suffering.

### Generation of Single, Double, and Triple MARP KO Mice

**Figure 1:**
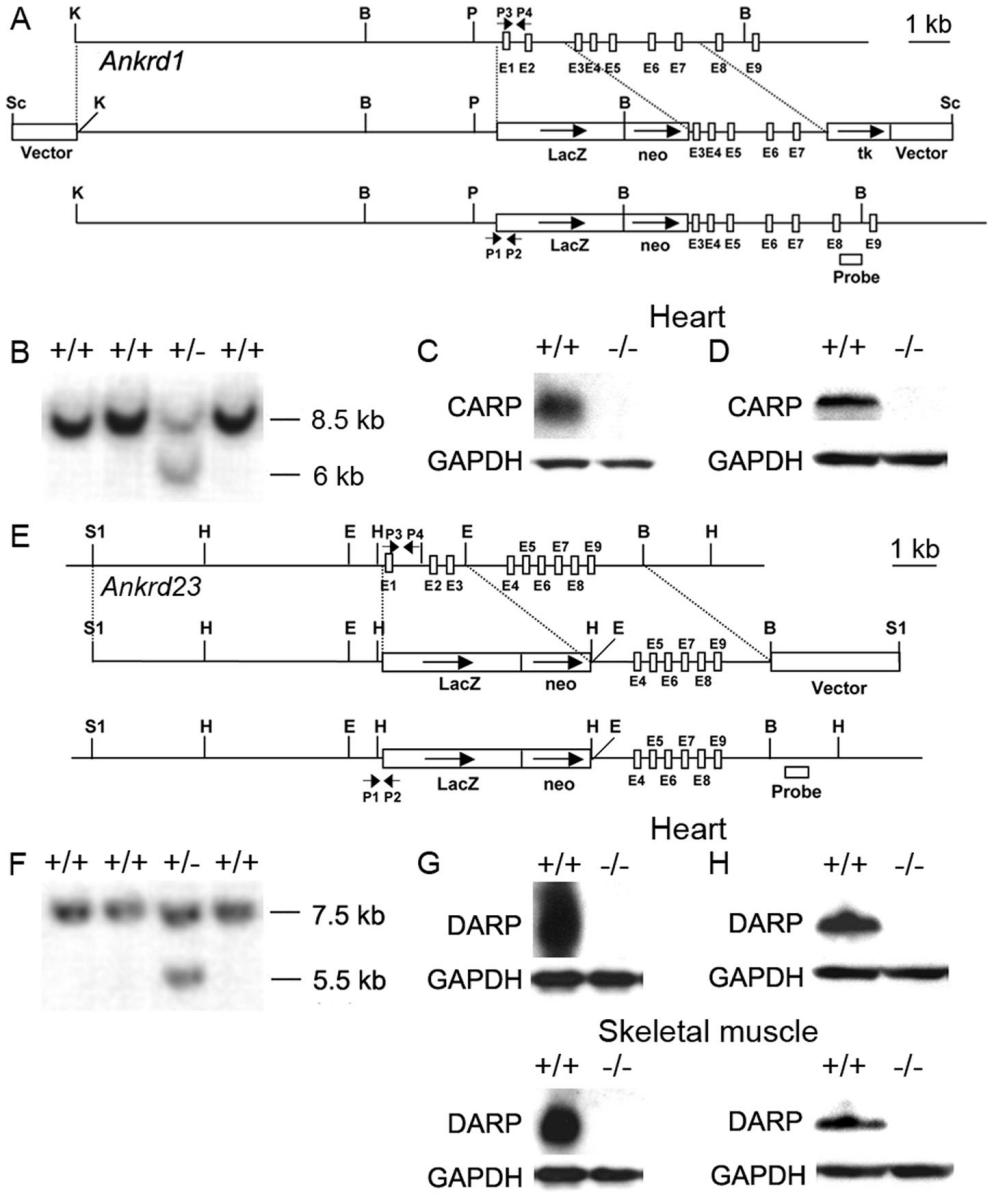
Generation of CARP and DARP KO mice. (A, E) Targeting strategies. Restriction maps of relevant genomic regions of *Ankrd1* (A) and *Ankrd23* (E) are shown on top, targeting constructs are shown in the center, and mutated loci after homologous recombination are shown at the bottom. B, BamHI; E, EcoRI; H, HindIII; K, KpnI; P, PstI; S1, SalI, neo, neomycin resistance gene; TK, thymidine kinase, E, Exon, P, Primer. (B, F) Detection of WT and targeted alleles by Southern blot analysis. *Ankrd1* (B) and *Ankrd23* (F) DNAs from electroporated ES cells were digested with BamHI and HindIII, respectively and analyzed by Southern blot analysis with probes as shown in A. (C, G) Detection of *Ankrd1* (C) or *Ankrd23* (G) mRNA by Northern blot analysis. Aliquots of 10 µg of total RNA isolated from adult ventricles of WT and KO mice were analyzed by using a cDNA probe spanning the entire coding regions of *Ankrd1* and *Ankrd23*, respectively. A GAPDH probe was used as a control for equal loading. (D, H) Detection of CARP (D) and DARP (H) protein by Western blot analysis. Protein prepared from adult ventricles of WT and KO mice were analyzed with polyclonal antibodies against CARP and DARP. Monoclonal antibodies against GAPDH were used as loading control.

Although the analysis of the skeletal muscle phenotype of MARP triple KO mice has been published [42], the generation of the KO mice has not previously been described in detail. Genomic DNA clones were isolated from a mouse 129/SvJ genomic DNA library (Stratagene, La Jolla, CA), using full-length cDNA of *CARP*, *Ankrd2*, and *DARP*, respectively. The first 2–3 exons of each gene were replaced by cDNA encoding lacZ and a pGK neo cassette. In this manner, the β-galactosidase cDNA was brought under the control of the endogenous promoters, while also ablating the endogenous *CARP*, *Ankrd2*, and *DARP* genes, respectively. The generation of Ankrd2 KO mice has been described previously [41]. The targeting constructs were verified by sequencing and linearized before electroporation into 129/SvJ-derived ES cells at the Transgenic Core Facility at the University of California, San Diego. ES clones were screened for homologous recombination by Southern blot analysis with probes as shown in Fig. 1A, E, and Bean *et al* [41]. Cells from two independent targeted clones from each construct were microinjected into C57BL/B6 blastocysts and transferred into pseudopregnant mice. Male chimeras resulting from the microinjections were bred with female Black Swiss mice to generate germ line transmitted heterozygous mice. These were subsequently intercrossed to generate homozygous mice. Offspring from intercrosses were genotyped by PCR analysis using mouse tail DNA and wildtype (WT) and mutant allele specific primers. The following primers were used: CARP WT (sense: ATAGACTCACGGCTGCCAACAT; reverse: CTCCATTTCTGAACTCCCCAGG), mutant (sense: TGGGATGACTCGCATTGCTGAG; reverse: AGATGAAACGCCGAGTTAACGC); Ankrd2/Arpp WT (sense: AACTTCGAAGATCCGCTCCTGG; reverse: CATCAATGATCTCACGTCGCAG), mutant (sense: CACACTGGACAGGCCTCTTTCC; reverse: AGATGAAACGCCGAGTTAACGC); DARP WT (sense: GCAGTTGGTAAGGATGTGAGGA; reverse: TCCAACAAGGGCAAACCTACAA), mutant (sense: CCTGGGCACAAGCTTTGTCCTT; reverse: AGATGAAACGCCGAGTTAACGC). Double and triple MARP KO mice were generated by interbreeding of single KO mice. To ensure that WT control mice had the same genetic background as double and triple MARP KO mice, also WT mice were generated from the crosses.

### Protein Isolation and Western Blot Analysis

Animals were anaesthetized by intraperitoneal injection of a mixture of ketamine (100 mg/kg) and xylazine (5 mg/kg) and sacrificed by cervical dislocation before removal of tissues. Total protein extracts were prepared from heart and skeletal muscle [43] and subjected to Western blot analysis using polyclonal antibodies against CARP and DARP (5 µg/ml, described in Miller *et al*. [8]). Gluceraldehyde-3-phophate dehydrogenase (GAPDH) antibody was used for normalization (1∶1000; Sigma-Aldrich).

### Histology

Dissected mouse hearts were fixed with 4% paraformaldehyde followed by dehydration and paraffin embedding. 10 µm sections were stained with hematoxylin and eosin or by Masson’s trichrome method.

### Cardiac Functional Studies

For transthoracic echocardiography, mice were anesthetized with 1% isoflurane and imaged as previously described [44]. For surgical procedures, mice were anaesthetized by intraperitoneal injection of a mixture of ketamine (100 mg/kg) and xylazine (5 mg/kg) and anesthesia depth was monitored by toe pinching. Cardiac hemodynamic parameters were evaluated in 8-week-old female and 17-month-old male mice by insertion of a 1.4 French Millar catheter-tip micromanometer catheter through the right carotid artery into the left ventricle [10]. Pressure was recorded both and baseline and following stimulation with graded doses of the β-adrenergic agonist dobutamine (0.75, 2, and 4 µg/kg/min) as previously described [10]. Transverse aortic constriction (TAC) was performed with a 27-gauge needle on 17 to 18-week-old male mice as described elsewhere [44]. 14 days after TAC, cardiac morphology and function was evaluated by echocardiography and the gradient for the arterial blood pressure between the constriction was measured by cannulation as described [44]. Only mice showing an adequate pressure gradient (>50 mmHg) were included in the analysis. SHAM operated mice were used as controls.

### Statistical Analysis

Data are indicated as average ± standard deviation. Statistical comparisons between WT and KO mice were done using the unpaired Students t-test. A P value of <0.05 is considered significant.

## Results

### Generation of CARP, Ankrd2, and DARP KO Mice

To study the functional role of the muscle ankyrin repeat family *in vivo*, mice deficient for each of the three family members, CARP, Ankrd2, and DARP KO mice were generated. The generation of Ankrd2 KO mice has been described elsewhere [41]. For each of the three genes, the targeting vector was generated by replacement of the first 1–3 exons by lacZ and the neomycin resistance gene (neo), thereby disrupting gene expression (Fig. 1A, E, and Bean *et al* [41]). Screening for homologous recombination in ES cells was performed by Southern blot analysis with primers as indicated in Fig. 1A, B, E, F, and Bean *et al* [41]. Correctly targeted clones were injected into C57/B6 blastocysts and implanted into the uterus of pseudopregnant mice. The resulting chimeras were mated to Black Swiss mice and gave rise to germline transmitted heterozygous mice, which were subsequently interbred to generate homozygous mice as determined by PCR. Successful ablation of each of the three genes was confirmed by Northern (Fig. 1C, G, and Bean *et al* [41]) and Western blot analyses (Fig. 1D, H, and Bean *et al* [41]) using polyclonal antibodies against CARP, Ankrd2, and DARP. In agreement with previously published data, Western blot analysis of cardiac and skeletal muscle lysates revealed highest expression of CARP in heart, Ankrd2 in skeletal muscle, and DARP in both heart and skeletal muscle (Fig. 1D, H, and Bean *et al* [41]).

### Generation of MARP Triple KO Mice

CARP, Ankrd2, and DARP KO mice were born at expected Mendelian ratios, were fertile, and survived till adulthood. KO mice were indistinguishable from wildtype (WT) littermate control mice and detailed analyses revealed normal cardiac morphology and physiology and no significant differences in heart/body weight ratios (see below). Since the three proteins are highly homologous in structure and are all expressed in the heart, we hypothesized that they may have overlapping functions and generated double and triple MARP KO mice by intercrossing single KO mice. WT mice generated from these crosses were used as controls to ensure that KO and WT control mice had the same genetic background. The successful ablation of all three proteins was confirmed by PCR as well as Northern and Western blot analyses (Fig. 1C, D, G, H, and Bean *et al* [41]).

### MARP Triple KO Mice have Normal Cardiac Morphology

MARP triple KO mice were born at expected Mendelian ratios and were indistinguishable from WT control mice. No statistical difference in heart/body weight ratios between MARP triple KO and WT mice was observed and cardiac histological analysis by hematoxylin and eosin and Masson’s trichrome staining showed no signs of hypertrophy, infarction, fibrosis, necrosis, calcification, or fat infiltration in MARP triple KO mice up to 17 months of age (data not shown).

### MARP Triple KO Mice have Normal Cardiac Function

Cardiac function was evaluated noninvasively by echocardiography at 4 and 17 months of age. As shown in Table 1 and 2, left ventricular chamber dimensions, fractional shortening, and heart rate were normal in MARP triple KO mice compared to WT control mice. Furthermore, hemodynamic function was evaluated in 8-week- and 17-month-old mice by cardiac catheterization in the absence or presence of graded doses of dobutamine, but no significant difference in contractile function was found between MARP triple KO and WT mice either at baseline or in the presence of the β-adrenergic agonist dobutamine (Table 3).

**Table 1:** Echocardiographic analysis on 17-month-old male MARP triple KO mice compared to WT at basal conditions.

	WT (n = 8)	tKO (n = 8)
**Body weight (g)**	54±2	44±7*
**Heart rate (bpm)**	540±37	558±44
**LVIDd (mm)**	3.97±0.18	3.83±0.41
**LVIDs (mm)**	2.26±0.27	2.09±0.38
**IVSd (mm)**	0.78±0.03	0.76±0.04
**IVSs (mm)**	1.29±0.08	1.30±0.13
**LVPWd (mm)**	0.78±0.03	0.78±0.04
**LVPWs (mm)**	1.41±0.14	1.33±0.11
**LV FS, %**	43.3±5.0	45.6±6.2
**LVM (mg)**	111.6±6.4	103.8±14.2
**LVM/BW (mg/g)**	2.08±0.08	2.38±0.40
**VCF (circ/s)**	9.37±1.68	9.88±1.60

All data are presented as mean ± standard deviation. WT, wildtype; tKO, MARP triple knockout; BW, body weight; LVID, left ventricular inner diameter; LVS, interventricular septum; LVPW, left ventricular posterior wall thickness; LV FS, left ventricular fractional shortening; LVM, left ventricular mass; VCF, velocity of circumferential fiber shortening; bpm, beats per minute; circ, circumference; d, diastole; s, systole. *P<0.01.

**Table 2:** Echocardiographic analysis of 4-month-old male MARP triple KO mice compared to WT before and after induction of cardiac hypertrophy by TAC.

	Before TAC		14 days after TAC	
	WT (n = 6)	tKO (n = 6)	WT (n = 6)	tKO (n = 6)
**Age (weeks)**	18.3±0	17.3±0.3	20.6±0.5	19.7±0.2
**Body weight (g)**	36.0±4.9	37.0±3.5	36.8±3.3	36.0±2.8
**Heart rate (bpm)**	484±81	537±55	429±42	440±65^§^
**LVIDd (mm)**	3.79±0.19	3.50±0.30	3.77±0.32	3.62±0.49
**LVIDs (mm)**	2.16±0.35	1.93±0.24	2.25±0.36	2.21±0.45
**IVSd (mm)**	0.70±0.07	0.68±0.13	0.84±0.12	0.86±0.14
**IVSs (mm)**	1.26±0.09	1.19±0.12	1.42±0.10*	1.36±0.08^§^
**LVPWd (mm)**	0.73±0.09	0.71±0.08	0.89±0.15	0.90±0.11^§§^
**LVPWs (mm)**	1.21±0.08	1.16±0.08	1.42±0.10**	1.36±0.08^§^
**LV FS, %**	43.0±7.6	44.8±5.9	40.6±5.3	39.4±4.7
**VCF (circ/s)**	8.2±1.5	9.7±2.0	6.3±0.9*	6.3±0.9^§§^
**LVMd (mg)**	92.4±8.0	79.0±18.4	117.5±13.7**	112.2±7.1^§^
**LVM/BW (mg/g)**	2.57±0.42	2.13±0.58	3.19±0.39*	3.12±0.44
**LV/BW (mg/g)**			3.96±0.44	3.62±0.77
**PG (mm Hg)**			83.8±13.2	76.9±13.2

See the legend to Table 1 for details and description of abbreviations. TAC, transverse aortic constriction; PG, Pressure gradient. Data comparison was carried out before and after TAC. *P<0.05 and **P<0.01 for WT TAC vs. WT baseline; ^§^P<0.05 and ^§§^P<0.01 for KO TAC vs. KO baseline.

**Table 3:** Hemodynamic properties following dobutamine stimulation in MARP triple KO mice compared to WT.

		8-week-old females		17-month-old males	
	DOB	WT (n = 9)	tKO (n = 8)	WT (n = 8)	tKO (n = 8)
**Heart rate (bpm)**	basal	356±34	373±34	333±31	366±67
	0.75 u	370±39	378±34	357±17	382±60
	2 u	386±48	403±38	380±27	409±67
	4 u	430±69	475±26	407±24	470±70
**LVP_max_ (mmHg)**	basal	96±16	91±11	101±17	106±17
	0.75 u	95±17	93±14	115±21	114±18
	2 u	100±14	101±16	134±21	128±19
	4 u	107±13	107±9	149±19	144±18
**dP/dt_max_ (mmHg/s)**	basal	7941±966	7400±1035	8086±1552	7673±1485
	0.75 u	8027±931	7746±1652	9082±1393	8263±1598
	2 u	9591±1016	9882±2198	11313±2335	10142±2235
	4 u	12163±1502	12759±1162	13901±2420	13786±3250
**dP/dt_min_ (mmHg/s)**	basal	−5163±617	−5237±495	−6479±1324	−6390±846
	0.75 u	−5386±441	−5413±765	−7445±1340	−6975±802
	2 u	−5927±653	−6279±1509	−9087±1761	−8425±1254
	4 u	−7069±573	−7371±1333	−11134±1572	−10732±1695
**EDP (mmHg)**	basal	5.4±8.0	3.1±3.6	1.6±1.2	2.9±3.1
	0.75 u	5.1±7.3	3.7±3.4	2.9±1.7	3.0±3.4
	2 u	4.9±5.9	3.6±3.5	3.8±1.9	3.2±3.7
	4 u	4.7±5.8	3.4±3.8	5.1±2.9	3.2±4.1
**Exp. Tau (ms)**	basal	13.1±1.4	12.4±1.4	11.5±1.4	11.6±2.0
	0.75 u	12.0±1.3	12.4±1.5	11.2±0.9	11.6±2.1
	2 u	12.0±1.0	12.0±2.0	10.5±1.6	10.6±1.5
	4 u	10.5±0.7	11.0±0.6	9.2±0.9	9.1±1.2

Values are mean ± standard deviation. WT, wildtype; tKO, MARP triple knockout; LVP_max_, maximum end-systolic left ventricular pressure; dP/dt_max_, maximum positive first derivative of LVP (contractility); dP/dt_min_, maximum negative first derivative of LVP (relaxation); EDP, end-diastolic pressure; Exp. Tau, experimental Tau; DOB: dobutamine; u: units.

### MARP Triple KO Mice Show a Normal Hypertrophic Response to Transverse Aortic Constriction

It is well known that CARP, Ankrd2, and DARP are upregulated in response to stress such as cardiac hypertrophy [5], [9], [10], [12], [15]. Therefore, to study the effect of MARP triple KO on mechanical pressure overload-induced hypertrophy, we performed transverse aortic constriction (TAC) on 4-month-old MARP triple KO mice and WT control mice. Cardiac function before and 14 days after TAC was evaluated by echocardiography and pressure gradients generated by the aortic constriction were measured. As shown in Table 2, similar pressure gradients were produced in both groups. Following TAC, left ventricular wall thickness and mass were increased by similar amounts in both groups (Table 2), indicating that the response to hypertrophic stimuli is unaffected in MARP triple KO mice.

## Discussion

To study the functional role of the MARP family members *in vivo*, we generated CARP, Ankrd2, and DARP KO mice. Since no phenotype was found in any of the individual KO mice, we hypothesized that the three highly homologous proteins have overlapping functions in the heart and generated MARP triple KO mice. However, in our detailed analyses, we were unable to detect any significant abnormalities in cardiac development and basal cardiac function up to 17 months of age and the hypertrophic response in response to acute pressure overload induced by TAC was similar between WT and triple KO mice. On the other hand, transgenic mice with cardiac specific overexpression of CARP were recently found to show attenuated cardiac hypertrophy and fibrosis in response to pressure overload and isoproterenol infusion, although the they were not protected against heart failure [22]. One explanation for this apparent discrepancy could be that the constitutive upregulation of CARP to non-physiological levels in the heart results in non-specific effects. Alternatively, since the MARP KO mouse models are constitutive KO mice, it is possible that compensatory mechanisms counteract the effect of the absence of the MARPs. To clarify this, it would be necessary to study the effect of conditional KO or overexpression of the MARP members in adult mice *in vivo.* Nevertheless, in light of the strong induction of MARPs in the heart in response to by various forms of stress, including cardiac hypertrophy and cardiac disease, as well as an accumulating body of evidence suggesting important roles of the MARP protein family in cardiogenesis, gene regulation, sarcomeric stress/stretch sensing, and disease, it is very surprising that the absence of all three MARP family members does not appear to have any effect on cardiac function *in vivo*, in particular since an increasing number of mutations in the *Ankrd1* gene have been identified in patients with dilated and hypertrophic cardiomyopathy [19], [20], [21]. The most likely explanation for this is that the identified Ankrd1 mutations have dominant negative effects by interfering with the binding of CARP to its many interaction partners, while complete absence of the MARPs has a less damaging effect and may be compensated for by other mechanisms. This is for example the case for knockout mice of the Z-line protein myotilin, which show no phenotype although mutations in myotilin are causative for limb girdle muscular dystrophy 1A [45].

The lack of a cardiac phenotype in MARP triple KO mice following 14 days of TAC does not exclude that the MARP KO mice may show a phenotype after long-term TAC or in response to other types of stress conditions, such as cardiac ischemia, myocarditis, and chronic β-adrenergic stimulation. Also, it is possible that MARPs might play a role in other organs, such as in skeletal muscle and the vasculature. We previously studied skeletal muscle function in MARP triple KO mice and found a significant increase in resting sarcomere length associated with the expression of longer titin isoforms [42]. Furthermore, MKO mice showed greater muscle injury following eccentric contraction exercise as indicated by a greater torque loss, and although they recovered normally, this was associated with increased expression of the regulatory genes MyoD and Muscle LIM protein (MLP), suggesting a role of the MARP family members in regulating muscle gene expression. In regard to the vasculature, CARP has been demonstrated to be a downstream target of TGF-β/Smad signaling in vascular smooth muscle cells and vascular endothelial cells [46], [47], and to be upregulated during arteriogenesis and angiogenesis, suggesting its involvement in these processes [48], [49]. In addition, a role of CARP in the induction of angiogenesis and neovascularization during wound healing has been proposed [46], [50]. Future studies of the MARP KO mice will reveal whether the MARPs may play important roles in the vasculature *in vivo*.
