# Correction: The Muscle Ankyrin Repeat Proteins CARP, Ankrd2, and DARP Are Not Essential for Normal Cardiac Development and Function at Basal Conditions and in Response to Pressure Overload

**DOI:** 10.1371/journal.pone.0118523

**Published:** 2015-03-06

**Authors:** The PLOS ONE Staff

The following grant number from the Telethon Foundation, Italy, to M.L.B. is missing from the Funding section: GGP12282. The complete, correct funding information is as follows: This work was supported by grants from the National Institutes of Health, National Heart, Lung, and Blood Institute (R01-HL66100 and R01-HL106968 to J.C.; and P01-HL46345 to K.R.C.); and the Telethon Foundation, Italy (S07006TELA and GGP12282 to M.L.B.; http://www.telethon.it/en/funding-research). J.C. is the American Heart Association (AHA) Endowed Chair in Cardiovascular Research. The funders had no role in study design, data collection and analysis, decision to publish, or preparation of the manuscript.
